# Response to Zahid and Rehman

**DOI:** 10.1007/s00467-025-06871-6

**Published:** 2025-07-16

**Authors:** Barbora Piteková, Ivan Hric, Jakub Zieg, Eva Baranovičová, Patrik Konopásek, Jakub Gécz, Paul J. Planet, Viktor Bielik

**Affiliations:** 1 Department of Pediatric Emergency Medicine, National Institute of Children’s Diseases, Bratislava, Slovakia; 2 Department of Pediatric Urology, Faculty of Medicine, Comenius University and National Institute of Children’s Diseases, Bratislava, Slovakia; 3 Department of Pediatrics, Slovak Medical University in Bratislava, Bratislava, Slovakia; 4 Biomedical Center, Institute of Clinical and Translational Research, Slovak Academy of Sciences, Bratislava, Slovakia; 5 Department of Biological and Medical Sciences, Faculty of Physical Education and Sport, Comenius University, Bratislava, Slovakia; 6 Department of Pediatrics, Second Faculty of Medicine, Charles University, Prague, Czech Republic; 7 Biomedical Center Martin, Jessenius Faculty of Medicine in Martin, Comenius University in Bratislava, Martin, Slovakia; 8 Department of Children and Adolescents, Third Faculty of Medicine, Charles University and University Hospital Kralovske Vinohrady, Prague, Czech Republic; 9 Children´S Hospital of Philadelphia, Philadelphia, PA USA; 10 Department of Pediatrics, University of Pennsylvania, Philadelphia, PA USA

Dear Editors,

We would like to thank the authors of the letter for their interest and praise [1] in response to our recent pilot study [2]. We wholeheartedly agree with the limitations noted by the authors, which all relate to the sample size constraints of a pilot study in a pediatric population. We particularly agree with the need for longitudinal and well-controlled studies that account for differences in diet, socioeconomic factors, and geography [3]. We also note that pediatric studies in particular are impacted by a number of variables that may be much less important in adults such as birth mode, breastfeeding or number of siblings/parity [3]. While the inclusion criteria allowed a wide age range (28 days to 18 years), the actual distribution in our sample was heavily skewed toward early childhood. More than half of the participants were under 3 years of age and the majority were younger than 6 years. Only a few children over 8 years of age were included and may be considered potential outliers; however, their small number limits their impact on the overall results. Key findings remained statistically significant even after adjusting for age in the regression models. We agree that future studies with narrower age stratification and larger cohorts will help further clarify these associations. Moreover, children have extremely dynamic changes in their microbiome over the course of their development that coincide with weaning and gradual introduction of new foods [4]. The arc of this development presents specific methodological challenges that are only beginning to be addressed. We join the letter authors in advocating for large, longitudinal microbiome/metabolomic studies that work toward precise strategies for improving the health of children.

## Data Availability

All data generated or analysed during this study are included in this published or previously published article.
